# The influence of physical division of tablets on the variability of release kinetics of gliclazide

**DOI:** 10.1007/s00706-018-2176-0

**Published:** 2018-03-03

**Authors:** Dorota Wójcik-Pastuszka, Anna M. Biedrawa, Dorota Haznar-Garbacz, Witold S. Musiał

**Affiliations:** 1 Department of Physical Chemistry, Faculty of Pharmacy, Wroclaw Medical University, Wroclaw, Poland; 2 Department of Drug Form Technology, Faculty of Pharmacy, Wroclaw Medical University, Wroclaw, Poland

**Keywords:** Gliclazide, Oral hypoglycemic agents, Tablets formulation, Kinetics, Pharmacopoeial dissolution test, UV–Vis spectroscopy

## Abstract

**Abstract:**

Tablets are often used in splitting process when the appropriated, registered dose is not available on the market or patients exhibit swallowing difficulties caused by the size of the tablet. The aim of the work was to assess the impact of physical division of tablets on the kinetics of in vitro gliclazide release from the intact and divided tablets. Gliclazide was released from prolonged release tablets containing 30 or 60 mg of the drug into a phosphate buffer, pH 7.4 and the amount of the drug in acceptor fluid was determined by UV–Vis spectrophotometry. The dissolution profiles were fit to zero- and first-order kinetics as well as to the Korsmeyer-Peppas equation. The largest discrepancy in the values of rate constants was obtained in the case of the release of gliclazide from intact and from splitting tablets using zero- and first-order kinetics. The values of the rate constants *k*_0_ obtained from the release of the drug from the intact tablets and from fragments with a dose of the drug of 30 mg were (4.2 ± 0.1) × 10^−5^ g min^−1^ and (5.8 ± 0.1) × 10^−5^ g min^−1^, respectively, and *k*_1_ were (2.3 ± 0.1) × 10^−3^ min^−1^ and (4.7 ± 0.6) × 10^−3^ min^−1^, respectively. These discrepancies were confirmed by the value of *f*_2_ coefficient that was 45.9. The results suggest that physical division of tablets accelerate the release of gliclazide from its prolonged form.

**Graphical abstract:**

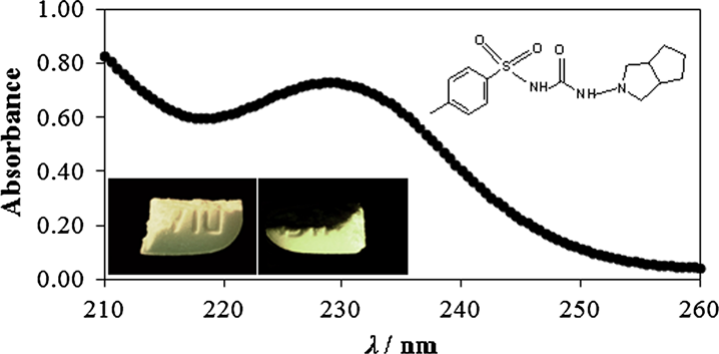

## Introduction

The oral dosage form, such as tablets is a very convenient method used in pharmacotherapy. Although there are a lot of tablets available in the pharmaceutical market, sometimes it is necessary to use a dose of a drug that is not available, especially in the case of children and older patients [1, 2]. Moreover, tablet splitting is used to facilitate the administration of formulations that cause swallowing problems, what is often associated with discomfort and can discourage patients from taking the medicine [3]. Another advantage of physical division of tablets is the economic aspect. The cost of tablets in terms of the amount of active substance often decreases with increasing the dose or the price is constant, independent of the dose. It is therefore preferable in some cases to purchase low amount of tablets with a higher dose that the patient can divide and reduce the cost of pharmacotherapy [4]. Despite the numerous advantages of tablets cutting there are also several disadvantages connected with splitting tablets. Not all tablets are suitable for dividing. Splitting of tablets can result in loss of modified release properties. These medications are formulated to release drug in a defined dosing period. Splitting process may result in an unintended large dose of the active substance or loss its efficacy [1]. It is common knowledge that tablets with a score line can be splitted. However, splitting tablets of a very small size or specific irregular shape can cause inaccuracies and generate losses in the mass. The lack of precision results in the unequal partition of the dosage form [5]. Cook et al. [6] studied the mass of cyclobenzaprine 10 mg tablets and theirs fragments obtained by dividing in two parts using a tablet splitter and a kitchen knife. It was revealed that fragments weight after splitting in a tablet splitter was in the range of 69.4–130.2% of the theoretical weight and the relative standard deviation (RSD) was 11.6%, corresponding to the drug amount between 3.47 and 6.51 mg. The weight of tablets parts obtained using kitchen knife was in the range of 49.9–149.5% with relative standard deviation (RSD) of 23.2%. The estimated drug content in this case was in the range of 2.49–7.48 mg. All these results were out of European Pharmacopoeia requirements that reported the amount of active ingredient to be in the range of 85–115% of the theoretical dose and RSD should be equal or smaller than 6%. Loss of mass upon breaking was established to not more than 1% [7]. It has been observed that the precision of tablet division is closely related to the method used for splitting. The most commonly method used for tablet’s breaking are manual division, kitchen knife or tablet splitter. The greatest weight loss was observed during manual division of tablets, especially in the case of older patients [8–11]. Habib et al. [12] studied the comparison between hand splitting parts of tablets and fragments obtained using tablet cutter. It was found that the smallest mass loss was observed in the case of the use of a tablet cutter. Uneven splitting tablets can cause fluctuations in the given dose. It is very important in the case of medicine with narrow therapeutic index such as: carbamazepine, cyclosporine, digoxin, ethosuximide, levothyroxine, lithium, phenytoin, procainamide, theophylline, warfarin, and tacrolimus [13]. Storing divided tablets that are removed from original package is another subject of study. It was reported that splitted tablets exposed to external conditions such as air, light, humidity can be unstable [14]. The weight variability, content non-uniformity and chemical degradation were found, especially in the case of digoxin formulations. Based on this observation it was stated that tablets should not be split ahead of time, but only immediately before administration.

The purpose of this work was to assess the influence of physical division of commercial tablets of gliclazide with prolonged activity, on the kinetics of the in vitro gliclazide release from the intact and splitted tablets.

## Results and discussion

The in vitro release study revealed that physical division of tablets results in fragments not always containing the amount of the drug that is necessary in pharmacotherapy. The mass of the tablet’s fragments obtained in present work, together with the amount of the drug and its percentage are listed in Table 1. It was noticed that the mass of gliclazide in all fragments obtained by halving prolonged release tablets containing 60 mg of gliclazide (T60) in two, were in the range 85–115% recomm vended by FDA [15]. However, in the case of splitting tablets with 30 mg of the drug (T30) in two, four from six fragments were out of the standard range, similarly as in the case of fragments arising from T60 divided into four parts. It should be mentioned that T60 have one score line, whereas T30 do not have any. These results suggest that the presence of a score line enables accurate division.

**Table 1:** The experimental mass of the tablet, its splitted fragments and the calculated mass of the drug

Series	Mass of the intact tablet/mg	Mass of the fragment/mg	Mass of the drug/mg	Percentage of the drug/%
30 mg obtained by halving a 60 mg tablet				
A	318.6	163.3	30.8	102.5
B		153.0	28.8	96.0
C	325.2	164.3	30.3	101.0
D		159.6	29.4	98.2
E	319.0	153.6	28.9	96.3
F		162.6	30.6	101.9
Mean ± SD	320.9 ± 3.7	159.4 ± 4.5	29.8 ± 0.8	99.3 ± 2.6
15 mg obtained by halving a 30 mg tablet				
A	159.8	67.2	12.7	84.7
B		92.4	17.4	116.0
C	159.5	60.2	11.3	75.3
D		99.3	18.7	124.7
E	157.9	68.5	13.0	86.7
F		89.3	17.0	113.3
Mean ± SD	159.1 ± 1.0	79.5 ± 14.7	15.0 ± 2.3	100.1 ± 18.5
15 mg obtained by dividing a 60 mg tablet in four				
A	320.4	97.8	18.3	122.1
B		54.8	10.3	68.4
C		68.5	12.8	85.5
D		90.6	17.0	113.1
E	320.7	111.3	20.8	138.8
F		58.9	11.0	73.5
Mean ± SD	320.6 ± 0.2	80.3 ± 9.5	15.0 ± 1.8	100.2 ± 11.8

**Fig. 1:**
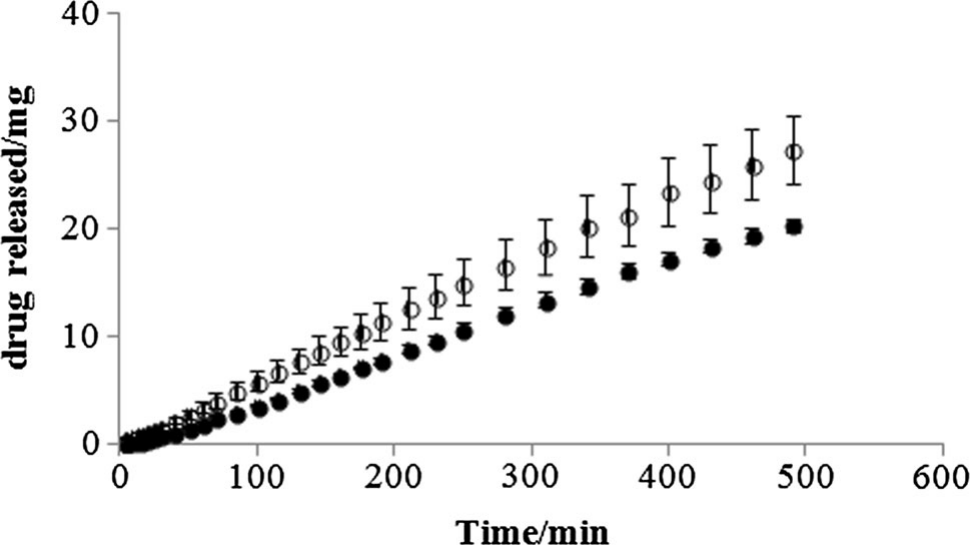
The dissolution profiles for the intact T30 tablets (filled circle) and T60 tablet fragments splitted in two (open circle)

The variability of gliclazide amount released in time is presented in Fig. 1. The mass of the drug released from six intact tablets T30 is almost the same and the standard deviation is very low in contrast to the mass of the drug released from fragments obtained by cutting T60 tablets using kitchen knife. The standard deviation is higher indicating that the amount of gliclazide released from each part is different. The dissolution and drug content of divided tablets affected by weight variability was also observed by Fahelelbom et al. [16]. It was found that among 40 tablets divided in half manually, 37 varied more than 10% from the mean weight and only 3 from 40 tablets divided in two using tablet splitter were did not comply.

The kinetic parameters of gliclazide release from prolonged release tablets obtained based on zero-, first-order kinetics as well as Korsmeyer-Peppas equation are listed in Table 2. The highest values of correlation coefficient *R*^2^ were derived in the case of zero-order model. It may be presumed that this model well describes the release of gliclazide from studied tablets. There was no difference between observed fitted kinetic models of the drug release, comparing intact tablets and from their fragments. The physical division of tablets does not influence the kinetic model.

**Table 2:** The kinetic parameters of gliclazide release from the intact tablets and formulations obtained after tablet splitting

Kinetic model	Kinetic parameters	Evaluated dose of					
		15 mg		30 mg		60 mg	
		Fragment of T30	Fragment of T60	Intact T30	Fragment of T60	Intact T60	Two intact T30
Z-O	*k*_0_ × 10^−5^/g min^−1^	3.0 ± 0.1	3.6 ± 0.1	4.2 ± 0.1	5.8 ± 0.1	9.3 ± 0.2	8.2 ± 0.1
	*R* ^2^	0.9934	0.9937	0.9981	0.9990	0.9979	0.9987
	*t*_0.5_*/*min	257.4 ± 7.7	223.3 ± 5.8	356.4 ± 5.8	260.3 ± 2.7	326.9 ± 5.0	367.5 ± 5.0
F-O	*k*_1_ × 10^−3^/min^−1^	4.5 ± 0.3	6.5 ± 1.0	2.3 ± 0.1	4.7 ± 0.6	2.6 ± 0.2	2.3 ± 0.1
	*R* ^2^	0.9703	0.8823	0.9812	0.9002	0.9734	0.9837
	*t*_0.5_*/*min	174.4 ± 9.7	113.7 ± 15.1	305.7 ± 16.2	163.1 ± 18.0	268.4 ± 16.6	312.8 ± 15.0
K-P	*k*_K–P_ × 10^−3^/min^−*n*^	1.4 ± 0.2	3.9 ± 0.5	0.5 ± 0.07	2.4 ± 0.2	1.2 ± 0.1	2.5 ± 0.2
	*n*	1.12 ± 0.06	0.92 ± 0.05	1.26 ± 0.05	0.98 ± 0.04	1.10 ± 0.02	1.01 ± 0.03
	*R* ^2^	0.9781	0.9788	0.9874	0.9899	0.9963	0.9933
	*t*_0.5_/min	216.9 ± 27.7	216.7 ± 29.4	243.4 ± 27.1	237.9 ± 23.2	244.8 ± 13.3	242.8 ± 17.2

*Z-O* zero-order, *F-O* first-order, *K-P* Korsmeyer-Peppas model

**Fig. 2:**
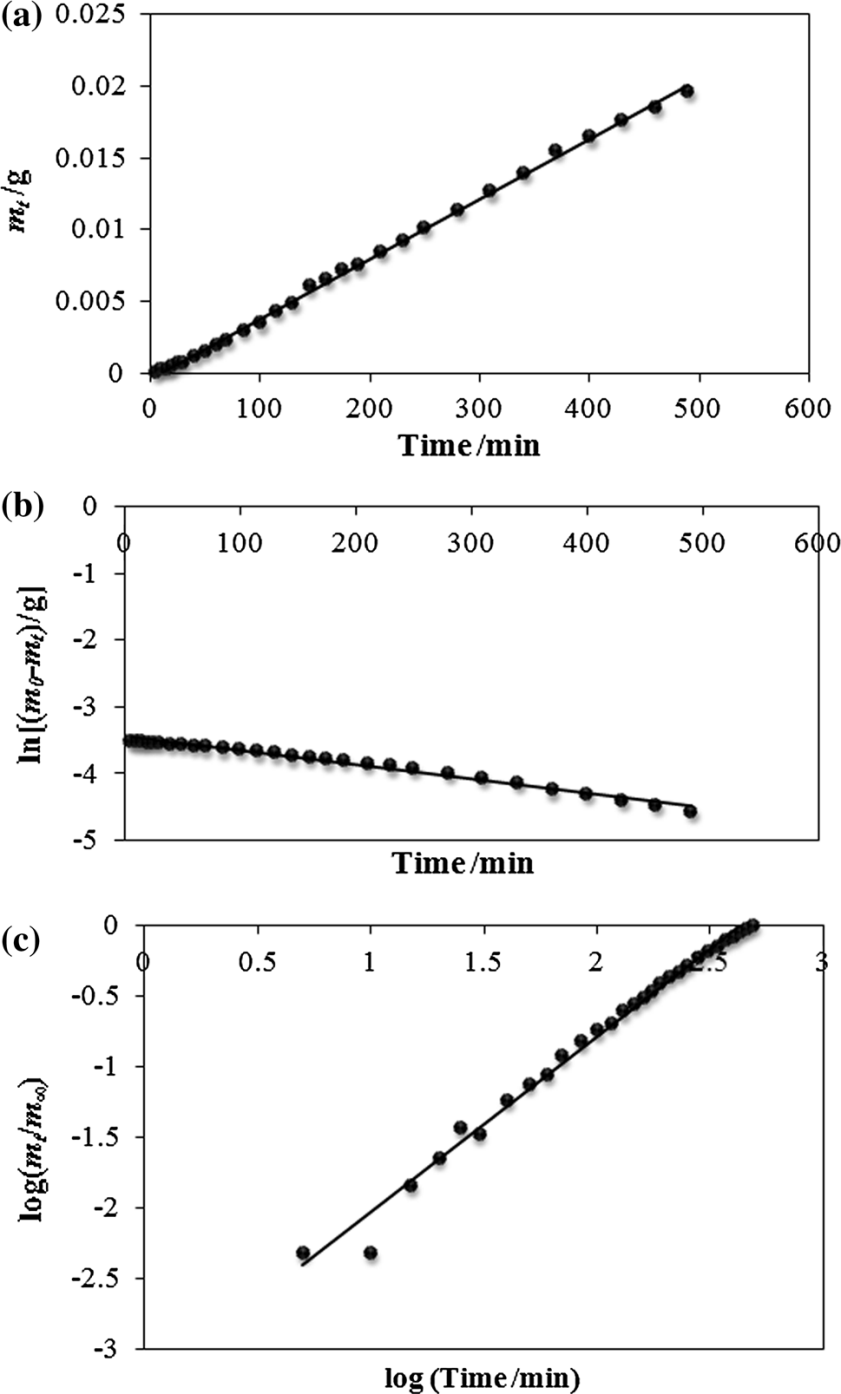
The kinetics plots of gliclazide release from intact tablets T30 tablet using **a** zero-order kinetics, **b** first-order kinetics, and **c** the Korsmeyer-Peppas model; experimental data: filled circle, solid line: linear regression

The rate constants *k*_0_ calculated on the base of zero-order kinetics for the initial dose of the drug of 15 mg are similar (3.0 ± 0.1) × 10^−5^ g min^−1^ (3.6 ± 0.1) × 10^−5^ g min^−1^, respectively, for the fragments obtained by splitting T30 in two and for fragments arised by splitting T60 in four. The values of *k*_0_ derived for the dose of 60 mg obtained from the intact tablet T60 and from two intact tablets T30 were (9.3 ± 0.2) × 10^−5^ g min^−1^ and (8.2 ± 0.1) × 10^−5^ g min^−1^, respectively. Slight discrepancies were noticed for the release of gliclazide from the dose of 30 mg. The values of *k*_0_ were (4.2 ± 0.1) × 10^−5^ g min^−1^ and (5.8 ± 0.1) × 10^−5^ g min^−1^ achieved for the release of gliclazide from intact tablets T30 and fragments formed by dividing tablets T60 in two, respectively. The values of the half release time *t*_0.5_ calculated according to zero-order kinetic model showed the greatest discrepancy also in the case of dissolution of gliclazide from the intact tablets T30 and fragments created by division of tablets T60 in two and were 356.4 ± 5.8 and 260.3 ± 2.7 min, respectively. The example of fitting experimental data to the theoretical curve of zero-order kinetics is presented in Fig. 2a.

Similar results were obtained from first-order kinetic analysis. In Fig. 2b experimental points analyzed using first-order kinetics are shown. It was revealed that the largest inconsistency between the value of the rate constants *k*_1_ was in the case of dissolution of gliclazide from intact tablets T30 and from fragments obtained by splitting T60 in two: (2.3 ± 0.1) × 10^−3^ min^−1^ and (4.7 ± 0.6) × 10^−3^ min^−1^, respectively. It was found out that also the half release time *t*_0.5_ shows the greatest divergence and its values were 305.7 ± 16.2 and 163.1 ± 18.0 min derived from the release of gliclazide from the intact and divided tablets T30 and T60, respectively.

According to the results of the analysis based on the Korsmeyer-Peppas model the biggest differences of the rate constants were found between the value (1.4 ± 0.2) × 10^−3^ min^−*n*^ and (3.9 ± 0.5) × 10^−3^ min^−*n*^ obtained for the release of gliclazide from T30 splitted in two and T60 divided in four, respectively. The values of half release time calculated using this model were the same within the error limits for each dose. The experimental data with the theoretical curve of Korsmeyer-Peppas model were presented in Fig. 2c. The value of the release exponent *n* was in the range from 0.92 ± 0.05 to 1.26 ± 0.05 indicating that gliclazide transport mechanism was followed super case II transport [17–19].

It is worth to mention that the values of the rate constants *k*_0_, *k*_1_ were higher when gliclazide was released from divided tablets, than from intact tablets. Moreover, comparing the values of *k*_0_, *k*_1_, and *k*_*K*–*P*_ it was noticed that these values were higher when gliclazide was released from T60 divided in four than T30 divided in two. To conclude, gliclazide was released faster from tablets fragments, compared to intact tablets. It can be explained that in the process of tablets dividing its matrix was exposed on acceptor fluid and a new and uneven surface was created. This observation was consistent with the results obtained by Ishitsuka et al. [20]. In their study of the effect of tablet division on the release rate of the drug, it was found out that the drug release rate was higher in the case of tablets fragments than intact tablets used in the dissolution test. The microscopic investigation revealed that the surface of the fracture was rough and had many hollows increasing the drug release rate.

**Fig. 3:**
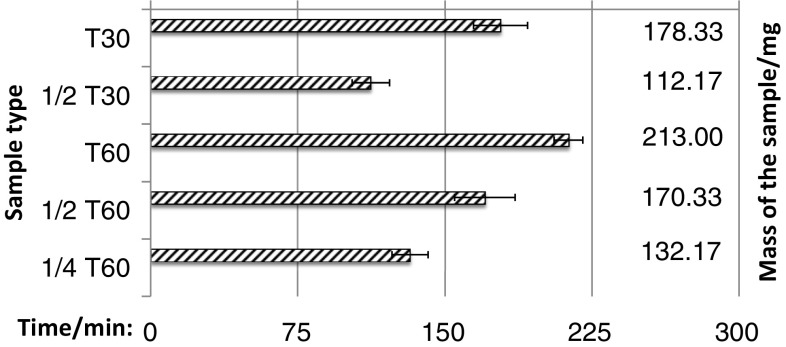
The mean time of disintegration of T30 and T60 tablets and their fragments obtained by physical division

The mean disintegration time of T30, T60, and theirs parts was in the range between almost 2 h (1/2 of T30), and over 3.5 h (T60). The differences were statistically significant only in one case—between intact T30 and intact T60. No statistical difference was observed between preparations containing same amount of gliclazyde, i.e.: T30 vs. 1/2 T60; 1/2 T30 vs. 1/4 T60 according to attached Fig. 3 and Table 3.

**Table 3:** The mean value of the weight and the disintegration time of T30 and T60 and their fragments obtained by physical division

	Evaluated dose of				
	15 mg		30 mg		60 mg
	Fragment of T30	Fragment of T60	Intact T30	Fragment of T60	Intact T60
Mean weight/g	0.076 ± 0.02	0.084 ± 0.008	0.159 ± 0.002	0.1605 ± 0.009	0.322 ± 0.002
Mean time of disintegration/min	112.2 ± 13.6	145.7 ± 46.1	178.3 ± 20.1	164.8 ± 18.1	213.0 ± 8.2

**Fig. 4:**
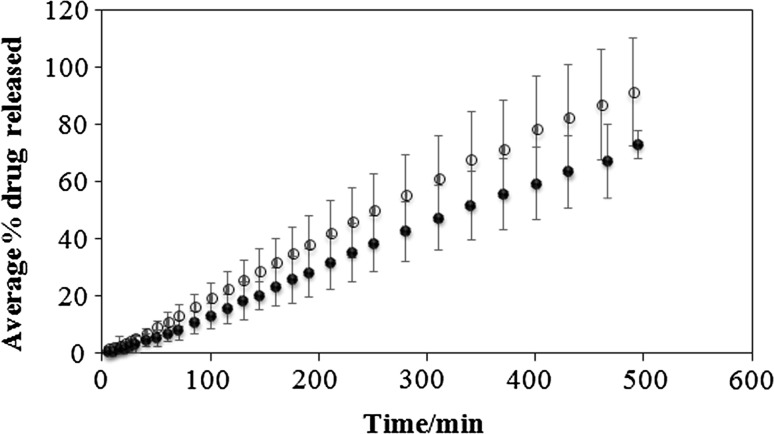
The in vitro release profiles of gliclazide from intact tablets T30 (filled circle) and from fragments obtained by dividing the tablets T60 in two parts (open circle)

The release profiles of gliclazide from tablets fragments and from intact tablets at the same dose of the drug were compared calculating the difference factor *f*_1_ as well as similarity factor *f*_2_ [15, 19]. Two dissolution profiles are considered similar when the factor of *f*_1_ is closer to zero (0–15) and the value of *f*_2_ is greater than 50. The obtained value of *f*_1_ and *f*_2_ are summarized in Table 4. It was noticed in this study that all values of *f*_1_ were below 15, meaning that there were no differences between the compared dissolution profiles of gliclazide. However, in the case of *f*_2_ the value of 45.87 was below 50 indicating the difference between the dissolution profile of gliclazide released from fragments of T60 divided in two parts and from the intact tablet of T30. The result was consistent with kinetics analysis. The largest discrepancy was observed between the rate constants of *k*_0_ and *k*_1_ derived for the release of gliclazide from fragments of T60 divided in two parts and from the intact tablet of T30. The dissolution profiles of gliclazide released from fragments of T60 divided in two parts and from the intact tablet of T30 are shown in Fig. 4.

**Table 4:** The difference factor *f*_1_ and similarity factor *f*_2_ values calculated for the mean in vitro dissolution profiles

Dose strength/mg	*f*_1_	*f*_2_
15	5.83	63.64
30	11.13	45.87
60	4.96	59.32

To compare the release profiles of gliclazide from tablets fragments and intact tablets, *t*-student test was carried out. In all cases no statistically significant differences were observed.

## Conclusion

To conclude, the in vitro dissolution study found out that the physical division of tablets, in particular without a dividing line, causes unequal fragments containing different dose of the drug than expected. The release of gliclazide from the studied formulations best describes zero-order kinetics and Korsmeyer-Peppas equationl. The physical division of T30 and T60 tablets does not change the release mechanism of the drug, although the discrepancy was observed between the rate constants of *k*_0_ (zero-order rate constant) and *k*_1_ (first-order rate constant) derived for the release of gliclazide from fragments of T60 divided in two parts and from the intact tablet of T30. The differences in the gliclazide release profiles were confirmed by the similarity factor *f*_2_ that was below 50.

## Experimental

Diaprel MR 30 mg tablets (Anpharm, Poland, Warsaw) and Diaprel MR 60 mg tablets Servier (France, Suresnes) were purchased in community pharmacy. Gliclazide was obtained from Sigma-Aldrich (USA, St. Louis). Sodium hydroxide and potassium dihydrogen phosphate anhydrous were delivered from Chempur (Poland, Piekary Śląskie). All chemicals were pharmaceutical grade and used without purification.

The in vitro investigation of gliclazide release was carried out from intact tablets T30 and T60 as well as from fragments obtained by splitting them with kitchen knife in two or four parts. All intact tablets and splitted formulations taken for the research were weighed using the analytical balance RADWAG (Poland, Radom).

The in vitro drug release study was carried out using USP paddle apparatus ERWEKA DT-700 (Germany, Heusenstamm). The dissolution vessels were filled with 1 l of phosphate buffer, pH 7.4 prepared according to recommendation of European Pharmacopoeia IX [21]. The experiment was performed at the temperature of 37 ± 0.2 °C and the rotation speed of 50 rpm. The 3 cm^3^ samples were withdrawn at fixed time intervals and fresh acceptor fluid was replaced at the same amount. The absorbance of collected samples was measured using UV–Vis spectrophotometer JASCO V-530 (Japan, Tokyo) at the wavelength of 229 nm [22]. The dissolution medium was used as a blank. The amount of the drug was calculated using the calibration curve. The dissolution study was conducted in six vessels for each batch of formulation. The obtained release profiles were analyzed with zero-, first- and second-order kinetics as well as Korsmeyer-Peppas model. The drug release profiles were assessed statistically using Student’s *t* test.

The dissolution conditions and apparatuses used for gliclazide release were tested by Skripnik et al. [23]. It was found that the in vitro release of the tablets was not depended on pH of the medium nor from the apparatus. However, the paddle apparatus was selected as the optimized—the paddle avoids the stickiness of formulations components at the basket mesh. It was observed that the rotation speed showed the greatest influence on the dissolution process. Almost 100% of the drug was released at 100 rpm and 71% was released in the case of 50 rpm.

The study of disintegration time of modified release tablets T30 and T60 and their fragments was performed using the basket apparatus Erweka ZT 51 (Germany, Heusenstamm) complied with the pharmacopoeial requirements. In the experiment all tablets and their fragments were analyzed six time, in the temperature 37.0 ± 2 °C, in phosphate buffer, pH 7.4 [24]. The study was carried out for the intact tablets of T30 and their fragments obtained by splitting them in two elements and for the intact tablets of T60 and their fragments arising by dividing them in two and in four parts. The total disintegration of the tablets or the fragments was considered as the end of the experiment.
